# LF-MF inhibits iron metabolism and suppresses lung cancer through activation of P53-miR-34a-E2F1/E2F3 pathway

**DOI:** 10.1038/s41598-017-00913-2

**Published:** 2017-04-07

**Authors:** Jing Ren, Liang Ding, Qianyun Xu, Guoping Shi, Xiaojing Li, Xiujun Li, Jianjian Ji, Dongya Zhang, Yaping Wang, Tingting Wang, Yayi Hou

**Affiliations:** 1 The State Key Laboratory of Pharmaceutical Biotechnology, Division of Immunology, Medical School, Nanjing University, Nanjing, 210093 China; 2 Jiangsu Key Laboratory of Molecular Medicine, Nanjing, 210093 China; 3 Department of Medical Genetics, Nanjing University School of Medicine, Nanjing, 210093 China

## Abstract

Our previous studies showed that low frequency magnetic fields (LF-MF) suppressed tumor growth and influenced the function of immune system. Nevertheless the mechanisms behind the effect of LF-MF still remain to be elucidated. In this study, Tumor- bearing mice subcutaneously inoculated with Lewis lung cancer cells were exposed to a LF-MF (0.4T, 7.5 Hz) for 35 days and Survival rate, tumor growth and the tumor markers were measured. Results showed that tumor growth was obviously inhibited with a prolonged survival of tumor- bearing mice by LF-MF exposure. *In vitro* experiments, LF-MF was found to induce cell growth arrest, cell senescence and inhibit iron metabolism of lung cancer cells. Moreover, LF-MF stabilized p53 protein via inhibiting cell iron metabolism and the stabilized p53 protein enhanced miR-34a transcription. Furthermore, increased expression of miR-34a induced cell proliferation inhibition, cell cycle arrest and cell senescence of lung cancer cells by targeting E2F1/E2F3. We also detected the relevant indicator in tumor tissue such as the iron content, the level of miR-34a and related protein, corresponding results were obtained. Taken together, these observations imply that LF-MF suppressed lung cancer via inhibiting cell iron metabolism, stabilizing p53 protein and activation P53- miR-34a-E2F1/E2F3 pathway.

## Introduction

Lung cancer is one of the most common causes of cancer-related morbidity and mortality, representing 13% of newly diagnosed cancers worldwide^1, 2^. Although radiotherapy and chemotherapy provide better therapeutic effects over the last decades, the toxicity and side effects are hard to tolerate for patients. The development of novel strategies for lung cancer is still critical^3, 4^.

Biological effect of magnetic fields (MF) on tumor development has been widely investigated^5, 6^. Epidemiological studies suggest that increased childhood leukemia risk is associated with residential magnetic fields^7^. While, most animal studies results that combined MFs with known carcinogenic agents have produce equivocal results and have not provide evidence of the enhancement of carcinogenesis by MF exposure^8, 9^. In a toxicity pilot human study, patients with heavily pre-treated advanced cancer treated with different schedules of time exposure to LF-MF and no toxicity and adverse side effects were observed^10^. Of note, LF-MF, with property of the non-invasive, non-ionizing and non-thermal effects on cells and tissues, has been used to study the influence of various diseases, including cancer, pain, and spasticity reduction^5, 11, 12^. LF-MF inhibited cell growth and induced cell apoptosis and cell cycle arrest of prostate cancer mediated by ROS *in vitro* ^13^. Several *in vivo* studies proved the anti-tumor effects of LF-MF with decreased tumor volume and longer survival time^14, 15^. Meanwhile, a 15-mT and 50-Hz LF-MF was introduced as a tumor necrosis agent^16^. A 5.5 mT and 50-Hz LF-MF was showed to have synergistic activity with chemotherapy (cisplatin) against lung cancer *in vivo* ^17^. Interestingly, LF-MF induced germ cell apoptosis while had no effect on prenatal development^18^. In our previous studies, we examined the inhibitory effect of LF-MF with different parameters on gastric carcinoma cells and chose 7.5 Hz as the suitable frequency of LF-MF in our magnetic field exposure system^19^. We also found that the LF-MF (0.4T, 7.5 Hz) inhibited the growth of gastric cancer, hepatocellular carcinoma and melanoma cancer cells and improved immune function in tumor-bearing mice^19–22^. However, the detailed anti-tumor mechanisms of LF-MF still need to be clarified.

MicroRNAs are a group of short single-stranded non-coding RNAs that exert biological functions by regulating transcription and/or translation of protein-coding genes^23^. miR-34a was reported to be down-regulated in several cancer cells including lung cancer^24–27^. The ectopic expression of miR-34a inhibited cell growth and induced apoptosis^26, 27^ and therapeutic delivery of miR-34a could inhibit lung tumor growth^25^.

Iron (Fe) is an essential element for all living organisms. It is involved in several fundamental biological processed^28^. Accumulation of iron in tissues increases the risk of cancer and TfR is frequently expressed multiple carcinoma cell lines^29^. The deficiency iron results in cell proliferation reduction and G1/S arrest of tumor cell. Depriving essential nutrient iron of cells by chelators has been used as an approach for cancer treatment^30^. Interestingly, previous study showed that LF-MF significantly changed iron concentration in liver and kidney^31^. However, to date it is not reported whether the interaction between iron and LF-MF may have an effect on cancer.

In this study, we found that LF-MF inhibited tumor growth in lewis lung cancer cells (LLC) mouse model. LF-MF also induced cell growth arrest and cell senescence in lung cancer cells. Specially, LF-MF enhanced the transcription of miR-34a and decreased the expression of E2F1/E2F3, which affect cell proliferation and cell senescence. We also confirmed that LF-MF suppresses the iron metabolism of lung cancer cells to stabilize p53 protein, which in turn enhance the transcription of miR-34a.

## Results

### LF-MF inhibits tumor growth in Lewis lung cancer murine model

**Figure 1:**
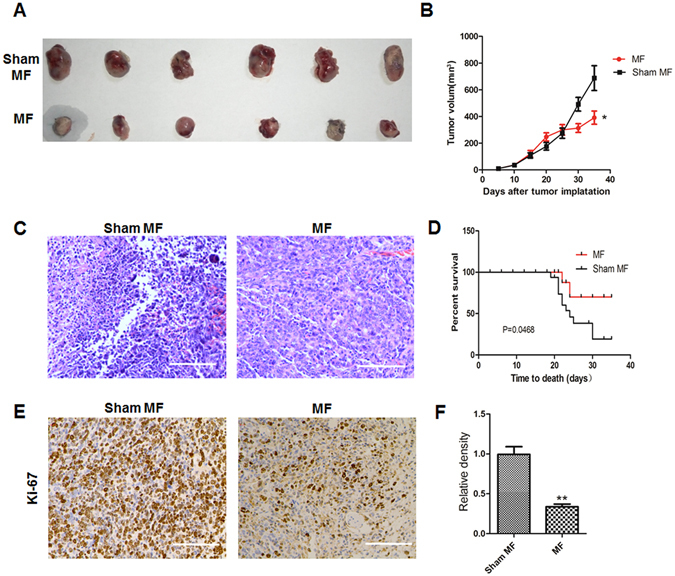
Low frequency magnetic fields inhibit tumor growth in a Lewis lung cancer murine model. C57BL/6 mice (n = 12 each group) subcutaneously inoculated with lewis lung cancer cells (5 × 10^5^) were exposed to LF-MF (0.4T, 7.5 Hz) or Sham MF for 35 days (2 h per day). (**A)** Representative images of LLC tumors after treatment of MF or Sham MF. (**B**) Growth curve of LLC tumors was calculated during treatment of MF or Sham MF. (**C**) Representative images of hematoxylin and eosin staining (10×) of tumors. (**D)** Survival curve of mice was calculated during treatment of MF or Sham MF. (**E**,**F**) Representative images of immunohistochemical (IHC) staining for Ki-67. Scale bars, 100 µm. (F) Positive cells of Ki-67 were counted using Image Pro Plus software 6.0. Data represent one of two independent experiments. Data represent Mean ± SEM. *P < 0.05, **P < 0.01 and ***P < 0.001.

We first examined the biological effects of LF-MF in Lewis lung cancer murine model. Mice inoculated with lewis lung cancer cells were exposure to Sham MF or LF-MF (0.4T, 7.5 Hz) for 35 days. Compared with Sham-MF group, tumor growth in LF-MF group was significantly decreased (Fig. 1A and B). Notably, mice in LF-MF group demonstrate prolonged survival compared with mice in Sham MF group (Fig. 1C). Histological analysis showed less intratumoral leukocyte populations in LF-MF group than Sham MF group (Fig. 1D). Immunohistochemical staining of Ki-67 showed less Ki-67 positive cells in LF-MF group compared with the sham MF group (Fig. 1E and F). These results suggest that LF-MF may be a potential therapeutic strategy to inhibit lung cancer.

### Effects of LF-MF on proliferation, cycle arrest and senescence of lung cancer cells

**Figure 2:**
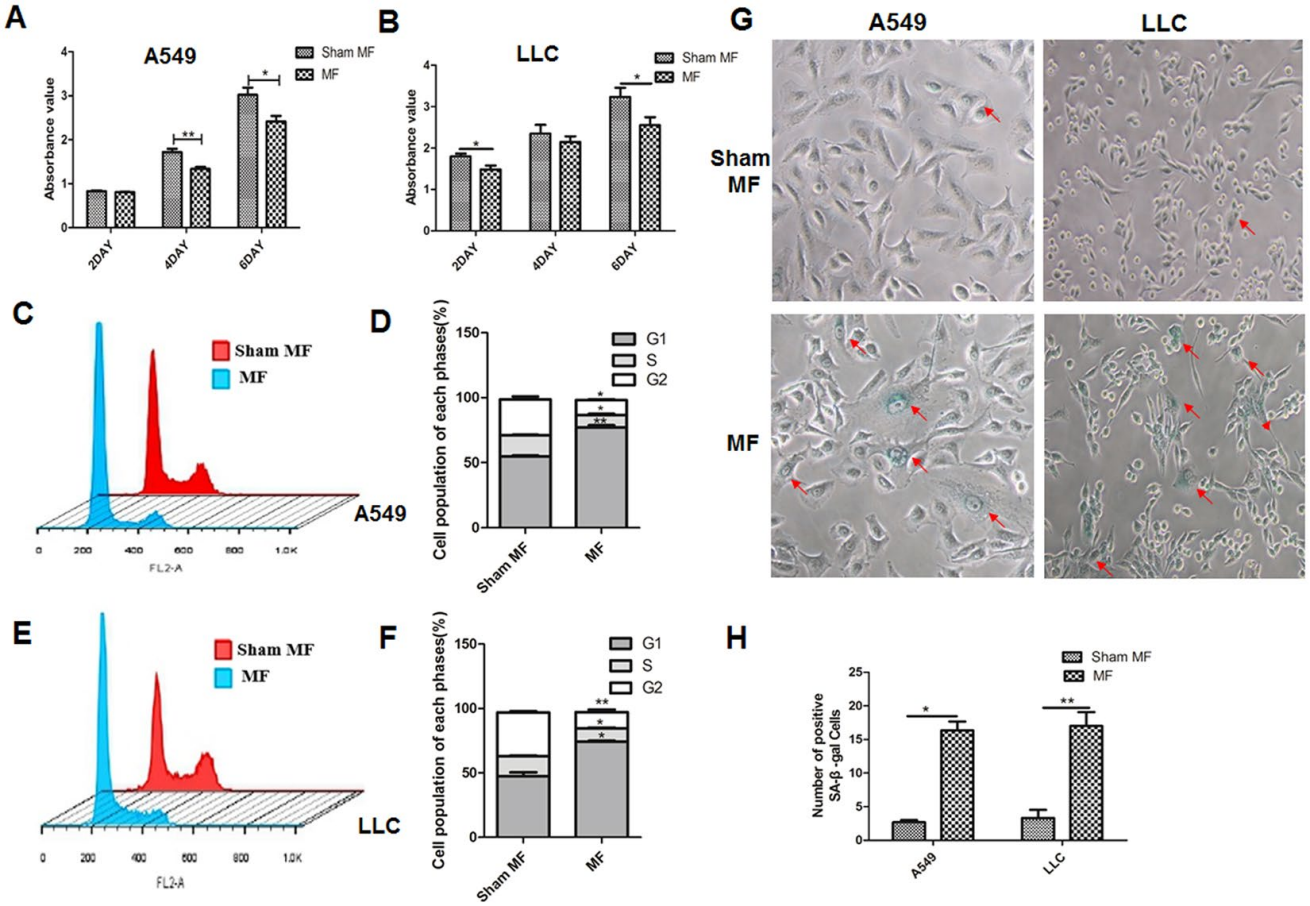
Low frequency magnetic fields induce cell proliferation inhibition, cell cycle arrest and cell senescence of lung cancer. A549 cells and LLC cells were exposed to LF-MF (0.4T, 7.5 Hz) or Sham MF for 6 days (2 h per day). (**A**,**B**) Cell proliferation rate was detected using CCK-8 assay after exposure to MF. **(C**,**D**) Cell cycle of A549 cells was detected by using flow cytometry after exposure to MF for 6 days. Mean percentage of G1, S and G2 phase cells was calculated. (**E**,**F**) Cell cycle of LLC cells was detected by using flow cytometry after exposure to MF for 6 days. Mean percentage of G1, S and G2 phase cells was calculated. (**G**) Cells was subjected to SA-*β*-gal staining after exposure to MF for 6 days. (**H**) Numbers of SA-*β*-Gal positive cells was calculated. All experiments were repeated three times. Data represent Mean ± SEM. *P < 0.05, **P < 0.01 and ***P < 0.001.

Our previous study had proved that LF-MF could inhibit proliferation of gastric cancer cells, lung cancer cells and colon cancer cells *in vitro* ^19^. Here, in consistent with previous data, the proliferation of A549 cells and LLC cells were inhibited by exposure to LF-MF (Fig. 2A and B). Cell cycle progression is an essential process by which cell monitors its growth and differentiation. Compared to Sham MF, A549 cells exposed to LF-MF displayed strikingly decreased number of cells in the S and G2/M phases and increased arrest in G0/G1 phase from 53.88% to 77.90% (Fig. 2C and D). A similar phenomenon was observed in LLC cells exposed to LF-MF for 6 days (Fig. 2E and F). Furthermore, after exposure to LF-MF for 6 day, the morphology of cells partially changed and became hypertrophic, suggesting the appearance of senescence. Staining of senescence-associated β-galactosidase (SA-*β*-gal) in lung cancer cells showed that LF-MF treatment up-regulate frequency of *β*-galactosidase (*β*-Gal)-positive cells (Fig. 2G and H).

### LF-MF up-regulates miR-34a to inhibit lung cancer growth

**Figure 3:**
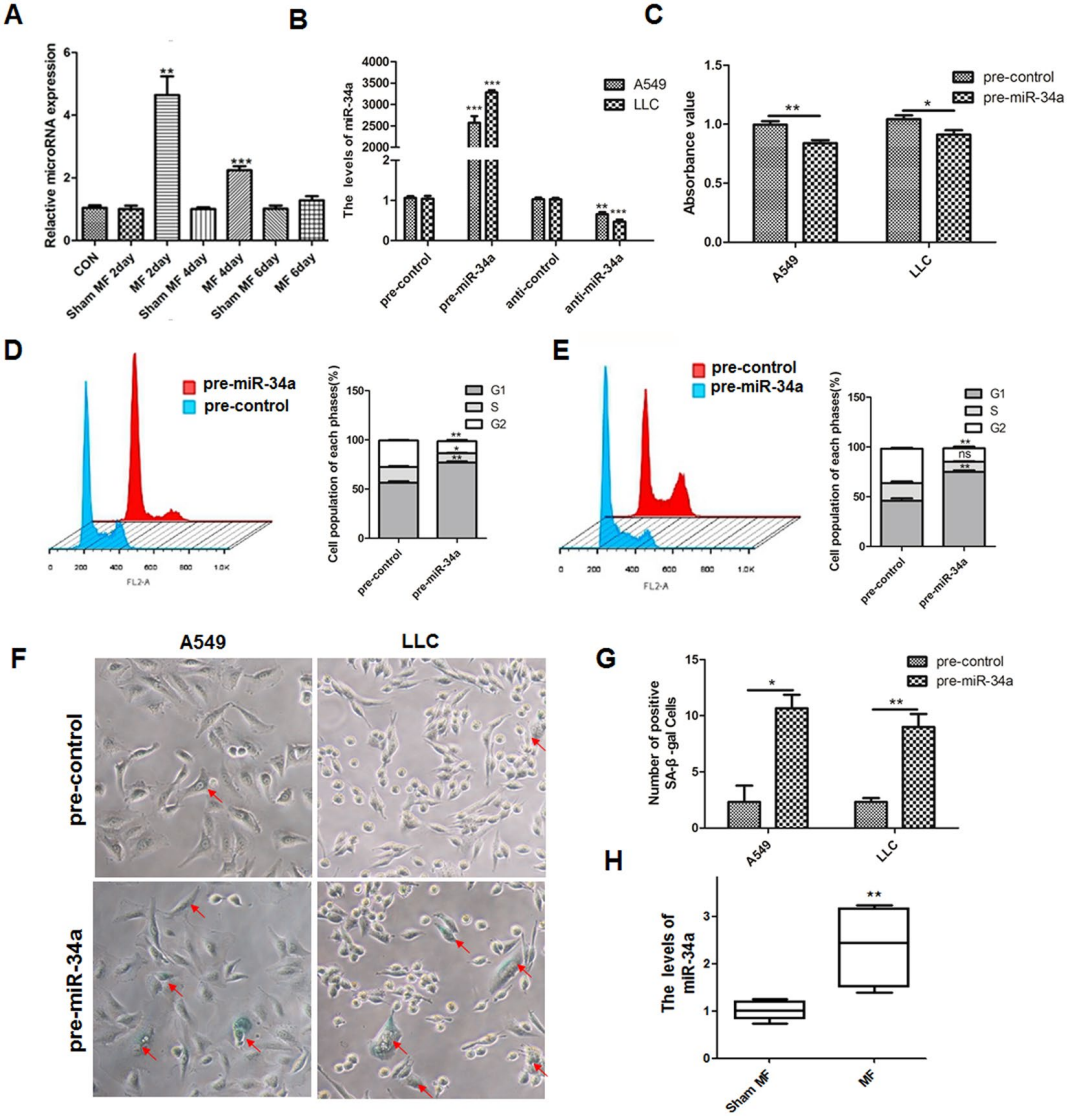
Low frequency magnetic fields regulate transcription of miRNA-34a. (**A**) Expression level of miR-34a in LLC cells was detected using qPCR after exposure to Sham MF or MF for 2, 4 and 6 days. (**B**) Q-PCR analyzes levels of miR-34a in LLC cells transfected with synthetic pre-miR-34a, pre-control, anti-control and anti-miR-34a for 48 h. **(C)** Cell proliferation analysis of cells transfected with synthetic pre-miR-34a and pre-control using CCK-8 assay. (**D**,**E**) Cell cycle analysis of A549 and LLC cells transfected with pre-miR-34a and pre-control for 48 h using flow cytometer. (**F**,**G**) Cells treated with MF were stained with SA-β-Gal. Numbers of SA-β-Gal positive cells was calculated. (**H**) Q-PCR analyzes levels of miR-34a in tumor tissue of LLC mouse model (n = 6). All experiments were repeated three times. Data represent Mean ± SEM. *P < 0.05, **P < 0.01, ***P < 0.001.

Recent study identified a significant change of 55 miRNAs with exposure to LF-MF *in vitro* ^32^. Upon checking several famous miRNAs related to cell proliferation, cell cycle and cell senescence, we found miR-34a level was up-regulated to 4.64 folds after 2 Days exposure to LF-MF (Fig. 3A). Here, LLC and A549 cells were transiently transfected with synthetic miR-34a precursor (pre-miR-34a), or antisense miR-34a inhibitor (anti-miR-34a) (Fig. 3B). Compared with negative control, ectopic expression of miR-34a significantly inhibited the proliferation (Fig. 3C) and induced cell G1 phase arrest in A549 cells and LLC cells (Fig. 3D and E). Moreover, the transfected A549 and LLC cells with pre-miR-34a appeared a senescence-like phenotype, enlarged cellular size and increased numbers of SA-β-gal positive cells (Fig. 3F and G). In consistent, LF-MF also could increase the expression of miR-34a in tumor tissue of LLC mouse model (Fig. 3H).

### MiR-34a inhibits lung cancer growth via modulation of the E2F pathway

**Figure 4:**
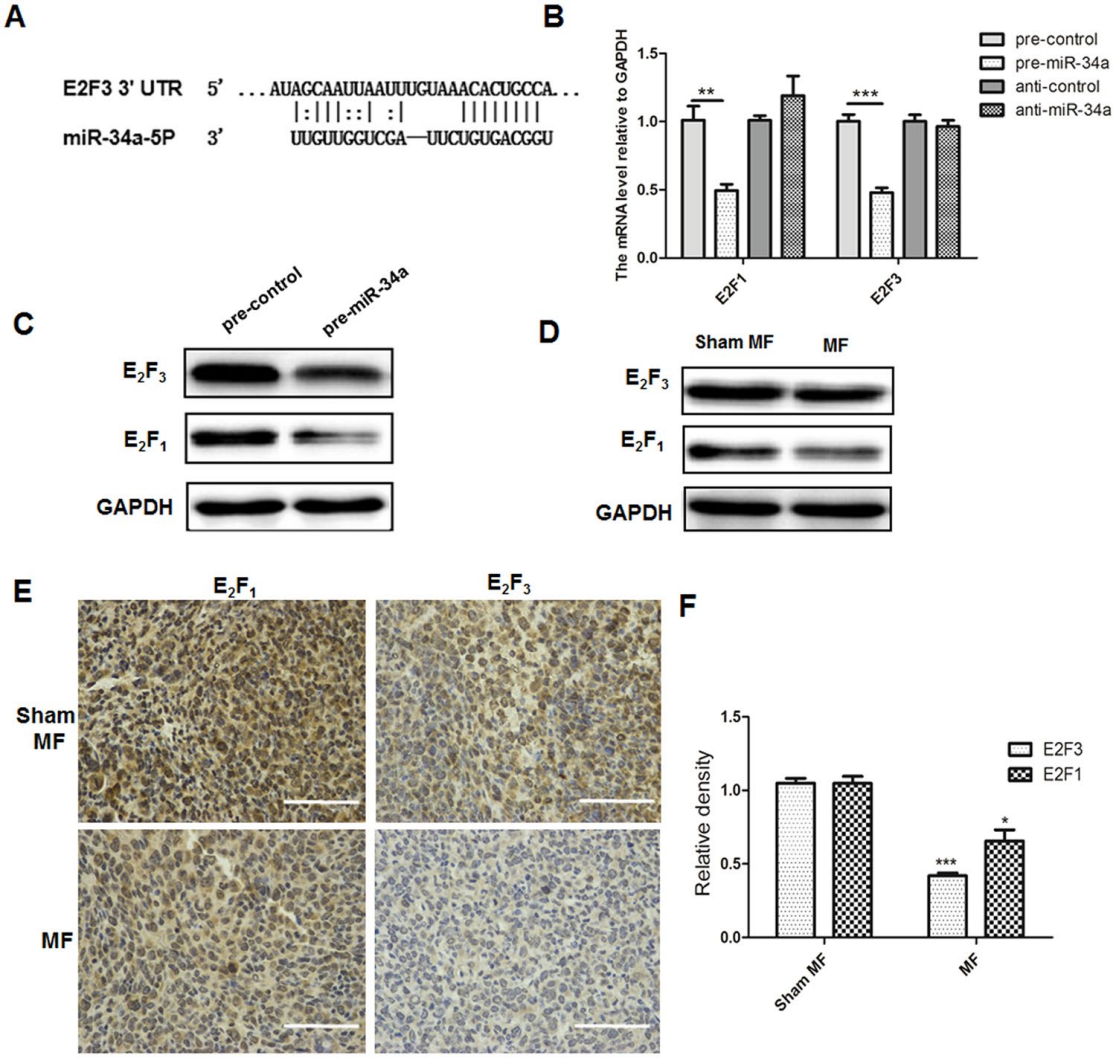
MiR-34a inhibits lung cancer growth via modulation of the E2F pathway. (**A**) Predicted miR-34a target sequence in the 3′UTR of E2F1 and E2F3. (**B**) Q-PCR analyzes the mRNA levels of E2F3 and E2F1 in LLC cells transfected with synthetic pre-miR-34a, pre-control, anti-control and anti-miR-34a for 48 h. (**C**) Western Blot analyzes E2F3 and E2F1 expression in in LLC cells transfected with pre-miR-34a and pre-control for 48 h. The numbers under each blot are relative intensity of the blot. (**D**) Western Blot analyzes E2F3 and E2F1 expression in LLC cells after treating with MF and Sham MF for 6 days. Relative intensity of the blot was added under each blot. (**E**) E2F3 and E2F1 expression in tumor tissue of LLC murine model was detected using IHC staining; Scale bars, 100 µm. (**F**) IHC images were calculated using Image Pro Plus software 6.0. All experiments were repeated three times. Data represent Mean ± SEM. *P < 0.05, **P < 0.01, ***P < 0.001.

MiR-34a was previously reported to induce senescence-like growth arrest through modulation of the E2F1/E2F3 expression in human colon cancer cells and leukemic cells^33^. Target prediction algorithms using targetscan (www.targetscan.org) or DianaLab (http://diana.cslab.ece.ntua.gr) identified a potential binding site for miR-34a in 3′-UTR region of E2F3, but not in E2F1(Fig. 4A). Previous studies reported that miR-34a regulated the expression of E2F1 through an indirect mechanism^33–35^. We wondered whether E2F1 and E2F3 were regulated by miR-34a in LLC cells. Indeed, ectopic expression of miR-34a downregulates the expressions of E2F3 and E2F1 at the translational level and protein level (Fig. 4B and C). Importantly, LF-MF exposure could down-regulated both E2F1 and E2F3 protein level in LLC cells (Fig. 4D). Moreover, a significantly decreased level of E2F1 and E2F3 was observed in tumor tissue of LLC mouse model with exposure to LF-MF (Fig. 4E and F). The E2F family of transcription factors also plays a critical role in the control of cell cycle^36^. Taken together, these data demonstrate that LF-MF can inhibit lung cancer through up-regulation of miR-34a and down-regulation of E2F pathway in lung cancer.

### LF-MF stabilizes p53 protein thereby increases expression of miR-34a

**Figure 5:**
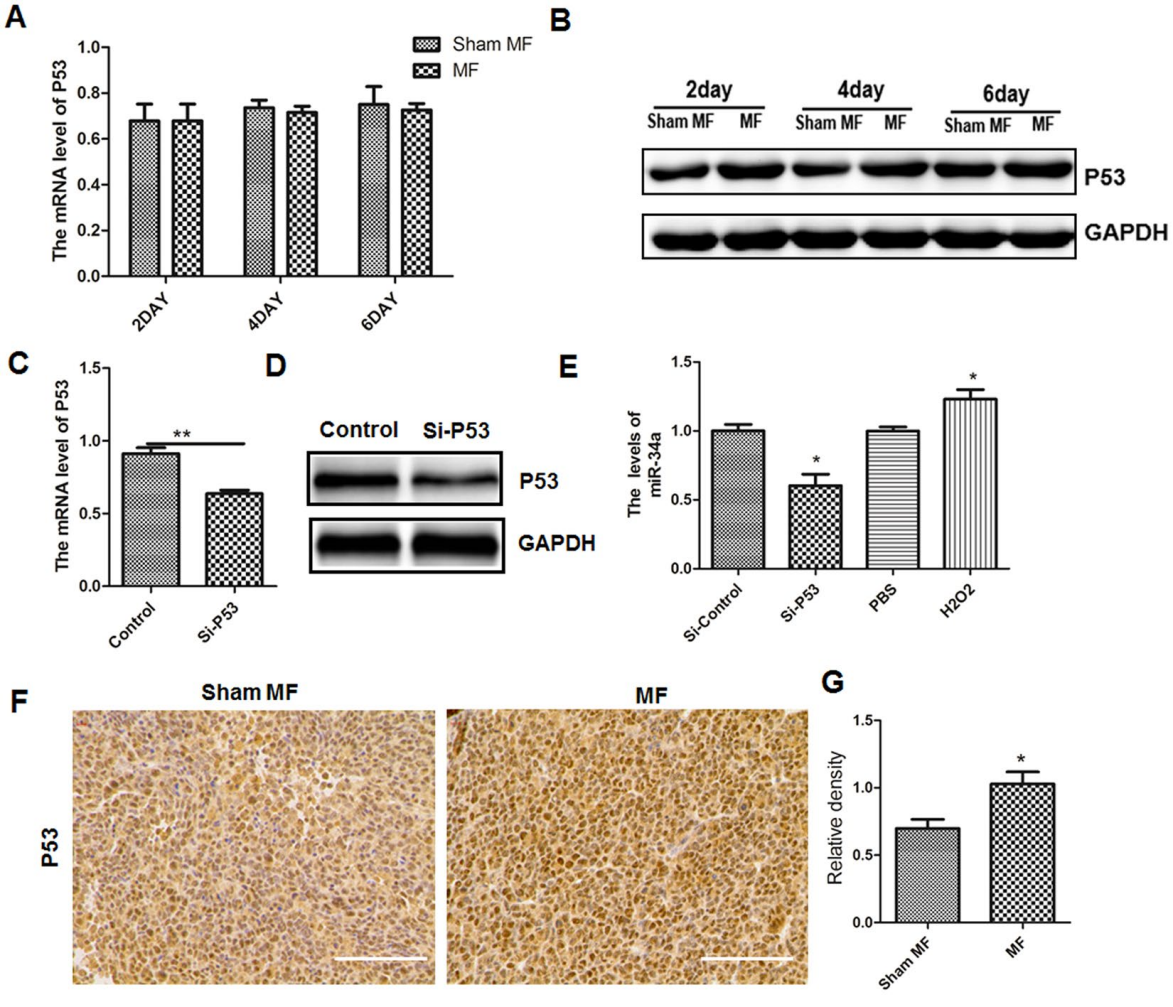
Low frequency MF up-regulated the expression of miR-34a by stabilizing p53 protein. (**A**) Q-PCR analyzes the mRNA levels of P53 in LLC cells treated with MF or Sham MF. (**B**) Protein level of P53 in LLC cells treatment with MF or Sham MF was detected using western Blot. (**C**) The mRNA levels of P53 in LLC cells transfected with siRNA-P53 was detected using Q-PCR. (**D**) Protein level of P53 in LLC cells transfected with siRNA-P53 was detected using western Blot. Numbers under each blot are relative intensity of the blot. **(E**) Q-PCR analyzes the miR-34a level after treatment of siRNA-TP53 and H2O2. (**F**) P53 expression in tumor tissue of LLC murine model was detected using IHC staining; Scale bars, 100 µm. (**G**) IHC images were calculated using Image Pro Plus software 6.0 and bar graphs. All experiments were repeated three times. Data represent Mean ± SEM. *P < 0.05, **P < 0.01, ***P < 0.001.

We next focus on how LF-MF regulates miR-34 expression. P53, which is a tumor suppressor gene, attended the proliferation of cancers and affected expressions of several miRNAs^37–39^. We therefore examined the mRNA level of P53 in LLC cells with exposure to LF-MF or Sham MF, respectively. No significant difference of P53 mRNA expression was found between Sham MF and LF-MF treatment (Fig. 5A). However, LF-MF treatment could up-regulate protein level of P53 in LLC cells (Fig. 5B). It is well known that the tumor suppressor protein p53 is unstable in quiescent cells and undergoes proteosomal degradation^40^. These data indicated that LF-MF might up-regulate the p53 protein level in LLC cells by stabilizing P53 protein.

To further confirm the relation between P53 and miR-34a, we analyzed the miR-34a expression level in LLC cells treated with siRNA-P53 (Fig. 5E). The transfection efficiency was verified by detecting mRNA and protein levels of P53 in LLC cells (Fig. 5C and D). Using PBS as negative and H_2_O_2_ as positive control, transfection of siRNA-P53 could decrease miR-34a level in LLC cells. Immunochemistry staining displayed higher levels of P53 in tumor tissue of LF-MF group than that in Sham MF group (Fig. 5F and G). Taken together, LF-MF could stabilize p53 protein thereby up-regulating the expression of miR-34a.

### LF-MF inhibits iron metabolism in lung cancer cells

**Figure 6:**
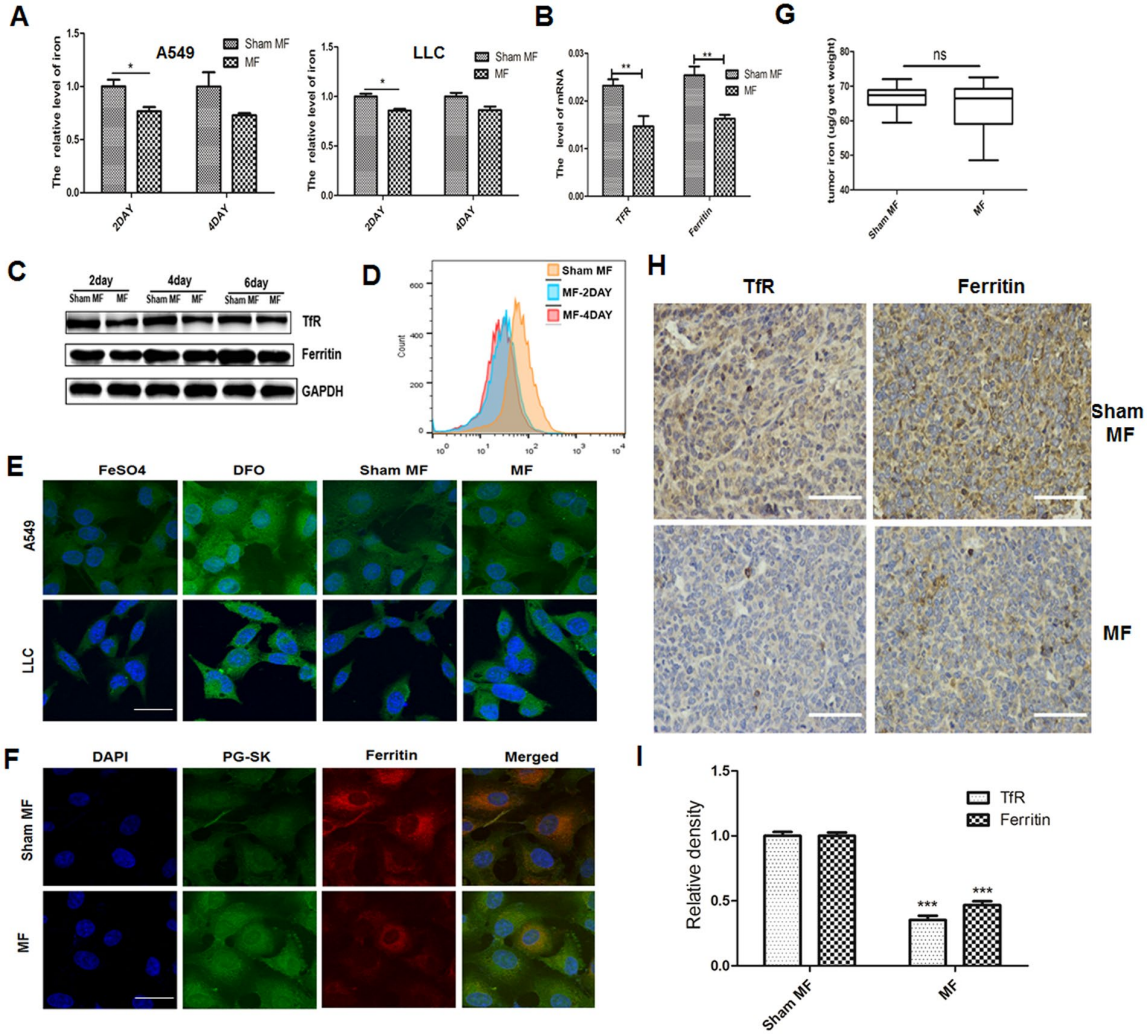
Low frequency magnetic fields induce lung cancer cell iron metabolism dysfunction. A549 and LLC cells were treated with MF or Sham MF for 2–4 days. (**A**) Cells were washed, digested with 5% HNO3 and the supernatant collected for intracellular non-haem iron estimation using flame atomic absorption spectrometer. (**B**) The mRNA levels of TfR and ferritin in LLC cells were detected using Q-PCR. (**C**) Protein level of TfR and ferritin in LLC cells were detected using western blot. Numbers under each blot are relative intensity of the blot. (**D**) Surface expression of TfR on LLC cells was detected using flow cytometry. (**E**) Immunofluorescence analysis of A549 and LLC cells treated with MF or Sham MF for 2days. Fluorescent probe Phen Green SK (PG-SK) was used for monitoring labile iron pool (Green). Cells treated with 100 μM ferrous sulfate (FeSO4) for 10 min were taken as positive control. Cells incubated with 100 μM DFO for 15 min were taken as negative control. Scale bars, 20 µm. (**F**) Immunofluorescence analysis of A549 cells treated by MF or Sham MF for 2 days. The cells were fixed and stained with ferritin antibody (red), PG-SK (Green), and DAPI (blue) respectively. Scale bars, 20 µm. (**G**) Measurement of non-haem iron content in tumor tissue of LLC murine model. (n = 6) (**H**) Immunochemistry analyzes TfR and ferritin-H expression in tumor tissue of LLC murine model. (n = 6); Scale bars, 100 µm. (**I)** IHC images were calculated using Image Pro Plus software 6.0. All experiments were repeated three times. Data represent Mean ± SEM. *P < 0.05, **P < 0.01, ***P < 0.001.

It has been shown that MF affected iron metabolism in tissues and organs^31, 41^. Interestingly, transferrin receptor, which can control iron metabolism, is frequently expressed in tumor cells^29^. To test whether LF-MF have effect on iron metabolism, we first evaluated the intracellular level of nonhaem iron in lung cancer cells. As shown in Fig. 6A, exposure to LF-MF for 2 days decrease intracellular iron pool in both A549 cells and LLC cells. As is well known, TfR1 binds to iron-loaded transferrin and plays a pivotal role in cellular uptake of iron as well as ferritin sequesters and stores the cellular iron. Intracellular iron concentration also regulates both the TfR1 and transferrin^42^. We then detected the mRNA and protein levels of both TfR1 and Ferritin in A549 cells. Compared with Sham MF, LF-MF exposure down-regulate the mRNA and protein levels of TfR1 and Ferritin (Fig. 6B and C). FACS assay showed that the surface expression of TfR1 also decreased after LF-MF treatment (Fig. 6D). To further confirm change of intracellular iron pool, labile iron pool was monitored *in situ* by using the fluorescent probe PG-SK, which can be quenched by binding intracellular labile iron. Cells treated with FeSO4 and iron chelator deferrioxamine (DFO) were used as positive and negative controls, respectively. Fluorescence was enhanced by exposure to LF-MF for 2 days both in A549 and LLC cells, indicating a decreased level of intracellular labile iron in lung cancer cells (Fig. 6E). It was reported that ferritin as the warehouse of excess intracellular iron storage can be regulated by intractellular labile iron level^43^. Immunofluorescence co-localization of intracellular ferritin and PG-SK showed that ferritin was decreased with reduced labile iron level after exposure to LF-MF for 2 days (Fig. 6F). Effect of LF-MF on iron metabolism was further confirmed in LLC murine model. Immunohistochemical analysis revealed decreased level of both TfR and ferritin in tumors of mice treated with LF-MF (Fig. 6H and I). In addition, no significant difference of total iron content in tumor tissues was found between Sham MF and LF-MF group (Fig. 6G). These data proved effect of LF-MF on iron metabolism in lung cancer cells.

### Inhibition of iron metabolism is requisite for LF-MF induced p53 protein stabilizing

**Figure 7:**
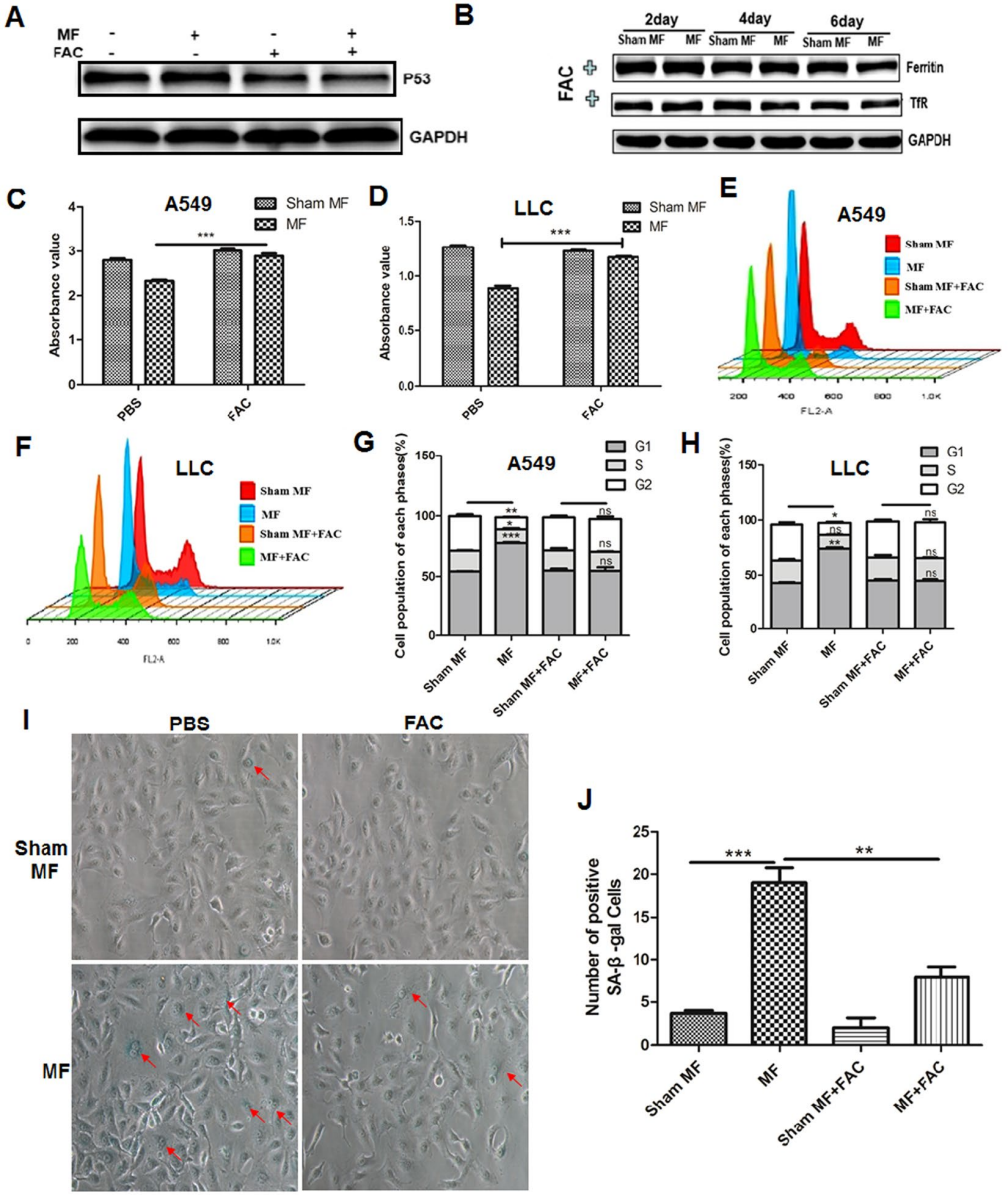
Low frequency MF stabilized p53 protein via inducing cell iron metabolism dysfunction. A549 and LLC cells were pre-incubated with medium containing FAC (100 uM) for 24 h and were exposure to MF or Sham MF for 6 days. (**A**) Protein level of P53 in LLC cells was detected using western blot. Numbers under each blot are relative intensity of the blot. (**B**) Protein expression of TfR and ferritin in LLC cells were detected using western blot. Numbers under each blot are relative intensity of the blot. (**C**,**D**) Cell proliferation of A549 cells and LLC cells was detected using CCK-8 assay. (**E**,**F**) Cell cycle of A549 and LLC cells were detected using flow cytometery. (**G**,**H**) Mean cell proportion in each phase was shown. (**I**,**J**) Representative image of SA-β-Gal staining assay was showed and numbers of SA-β-Gal positive cells was calculated. All experiments were repeated three times. Data represent Mean ± SEM. *P < 0.05, **P < 0.01, ***P < 0.001.

To explore whether P53 stability relates to iron metabolism, ferric ammonium citrate (FAC) was used as Fe supplementation^30, 44^. Western blot data showed that FAC reversed LF-MF induced increased level of P53 protein in lung cancer cells (Fig. 7A). Meanwhile, FAC significantly reversed LF-MF induced down-regulation of TfR1 and ferritin in A549 cells and LLC cells (Fig. 7B). The alterations of cell proliferation, cell cycle arrest and cell senescence with Fe supplementation were also evaluated. Although no change was observed in cell proliferation (Fig. 7C and D) and cell cycle between LF-MF and Sham MF group (Fig. 7E,F,G and H). The senescence-like phenotype with increased β-Gal-positive cells was eliminated after Fe supplementation (Fig. 7I and J).

## Discussion

**Figure 8:**
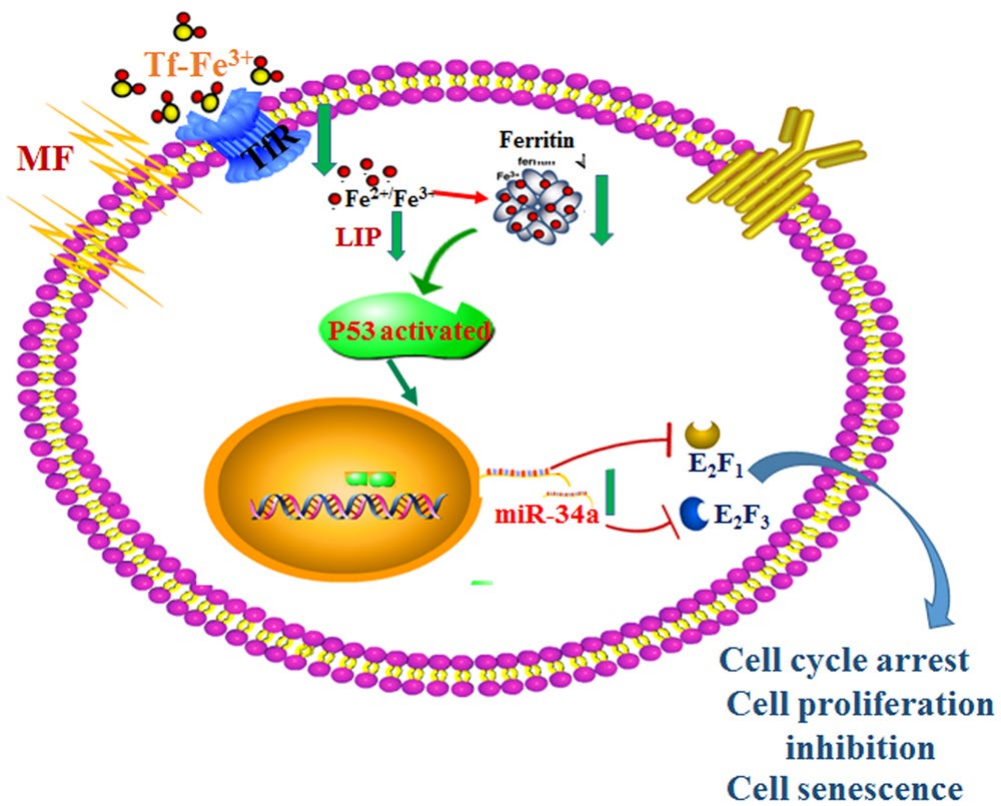
Working model of LF-MF induced cell proliferation inhibition, cell cycle arrest and cell senescence.

Although several studies explored the biological effects of LF-MFs on cancers, the molecular mechanisms remained elusive. In this study, we confirmed the anti-tumor effect of LF-MF in LLC murine model and provided an iron-p53-miR-34a-E2F1/E2F3 pathway to clarify the mechanism of LF-MF. Specifically, LF-MF inhibits iron metabolism and induces p53 protein stabilizing in lung cancers. LF-MF stabilizes p53 protein thereby increases expression of miR-34a. Meanwhile, E2F1/E2F3 are direct targets of miR-34a, which regulate cell proliferation and senescence (Fig. 8).

A significant change of iron and copper concentration in the liver and kidneys of fertilized rats exposed to static and LF-MFs have been observed^31^. Cellular iron metabolism is critical for many cellular processes, including oxygen transport, respiration and DNA synthesis^45^. Dysregulation of iron metabolism was found in most cancer cells. Cellular iron content must be tightly controlled, as iron deficiency can cause growth arrest and cell death, whereas iron in excess generates free radicals that damage DNA, lipid membranes and proteins^42^. We observed that the level of intracellular nonhaem iron and intractellular labile iron were significantly decreased after 2 days LF-MF exposure. Moreover, Ferritin as the warehouse of excess intracellular iron storage and TfR1 binds to iron-loaded transferrin for uptake of iron decreased both in mRNA and protein level. Consistently, we found that TfR and ferritin-H (FLH) was lower in tumor tissues of LLC mice model treated LF-MF. However, no statistically significant differences of total iron content of tumor tissues was found between Sham MF and LF-MF group. We suspected that this was because the alteration of intracellular iron had a short lifetime. P53 can induce a permanent inhibition of cell proliferation, through the induction of cell death, senescence and differentiation in different ways^46^. miR-34a, a member of miR-34a family (a, b and c) are identified as p53 target genes and plays an important role in p53-mediated processes, including cell cycle arrest, senescence and apoptosis^47^. Our study reveals that LF-MF induces cell proliferation inhibition, cell cycle arrest and cell senescence of lung cancer by P53-miR-34a-E2F1/E2F3 pathway activation. Exposure of LF-MF significantly increased miR-34a and up-regulated P53 protein level. The relation between P53 and miR-34a were further confirmed by analyzing the miR-34a expression level in LLC cells treated with siRNA-P53 and H_2_O_2_. We further confirmed that ectopic expression of miR-34a significantly inhibited the proliferation, induced cell G1 phase arrest and a senescence-like phenotype in LLC and A549 cells by targeting E2F1/E2F3. E2F1/E2F3, two members of E2F family, are major regulators of the cell cycle, apoptosis and differentiation, and function as transcriptional activators in humans. As a tumor suppressor and oncogene, the transcription factor E2F1 is a downstream regulator of the Rb pathway^48^. Global gene expression microarray analyses have showed that miR-34a inhibited cell proliferation by modulation of E2F signaling^33^. Previous study showed that DFO, a cellular iron-chelating agent, induced the accumulation of p53 protein in ML-1 cell^49^. Apart from essential for normal growth and development, p53 has an important role in determining the response of cells to numerous types of stress, including genotoxic stress, oncogene activation, ribosomal stress and a lack of oxygen or other nutrients^46^. 6mT static magnetic field was reported to induce apoptosis by increasing bax and p53 expression in freshly isolated human lymphocytes^50^. In Jurkat cells, 6 mT Static magnetic field could induce apoptosis and alter cell cycle via a p53-independent pathway^51^. The transcription levels of p53 was unaffected by 60 Hz magnetic fields exposure in human breast epithelial cells^52^. In accordance with our results, no significant difference of P53 mRNA expression was found between Sham MF and LF-MF treatment. Subsequently, we verify the hypothesis that LF-MF stabilized p53 protein via inhibiting cell iron metabolism in lung cancer cells. Further study will be necessary to clarify the specific molecular mechanism of iron metabolism inhibition of lung cancer induced by low frequency magnetic fields.

Together, LF-MF obviously suppressed the iron metabolism of lung cancer cells to stabilize p53 protein, which enhanced the transcription of miR-34a to inhibit lung tumor growth via targeting E2F1/E2F3. Our studies reveal a novel mechanism behind the effect of LF-MFs on lung cancer and provide a potentially useful adjunct therapy for treatment of lung cancer.

## Materials and Methods

### Experimental Magnetic Fields

The construction of instrument to generate experimental LF-MF has been described previously^19–21^. As shown in Supplementary Fig, two pairs of fan-shaped NdFeB permanent magnets (N45, Innuovo, Dongyang, China) were embedded into a circular iron plate and arranged to establish LF-MF. The bottom two magnets rotated at certain frequency driven by a step motor, which was controlled using a functional signal generator. Due to the strong magnetic interaction, the top two magnets rotated synchronously (Supplementary Fig. 1A). The flux density and frequency of LF-MF at the target site were alternative and the magnetic flux density was measured by gauss meter (HT201, Hengtong, Shanghai, China). 0.4T flux density and 7.5 Hz frequency was used in our study. Mice were placed in the middle of the magnets in a lucent and breathable box and could move freely in the box. There was an internal column in the center of the box, and mice were put between external column (D = 20 cm) and internal column (D = 8 cm). Control mice were placed in a similar apparatus except that there were two rotating iron plates instead of magnets, thus lacking a LF-MF. The entire magnetic apparatus was located in a hood with humidity and temperature controller. For cell experiment, a smaller instrument with similar structure to generate 0.4T and 7.5 Hz LF-MF was installed in Thermo Scientific Forma Series Water-Jacketed CO2 Incubators (Supplementary Fig. 1B). Control cells were placed in a similar apparatus except that there were two rotating iron plates instead of magnets (sham MF). The instrument was fabricated by the National Laboratory of Solid Microstructures, Nanjing University (Nanjing, China).

### Cell Culture and Treatment

Lung cancer cell lines A549 and LLC were obtained from the Cell Bank of the Chinese Academy of Sciences (Shanghai, China) and were cultured in RPMI medium 1640 (Gibco, Carlsbad, CA) supplemented with 10% fetal bovine serum (FBS) (Gibco, Carlsbad, CA) and 100 U/ml penicillin and 100 U/ml streptomycin (Amresco, Solon, OH).

### Lewis lung cancer (LLC) murine model

5 × 10^5^ LLC cells were injected into the right flank of female C57BL/6 mice (6–8 weeks). The tumor-bearing mice were exposed to a low frequency MF (0.4T, 7.5 Hz) 2 hours a day for 35 days. The tumor volume was calculated using the formula, V = (ab2)/2, where a is the long axis, and b is the short axis. All animal work was approved by the Animal Care Committee of Nanjing University in accordance with Institutional Animal Care and Use Committee guidelines.

### Determination of intracellular nonhaem iron content

The cellular nonhaem iron content was determined by atomic absorption spectrometry with acetylene-air flame atomization as described^53^. Briefly, Cells were washed with PBS and digested with 5% HNO3 at 60 °C for 2 h and supernatant collected for iron estimation using flame atomic absorption spectrometer (Spectra AA-6800 Spectrometer, Shimadzu, Japan).

### Measurement of cytosolic labile iron pool

Intracellular labile iron levels were monitored *in situ* by using the fluorescent probe Phen Green SK (PG-SK) (Invitrogen, Darmstadt, Germany), the protocol described by Petrat *et al*.^54^. For saturating the intracellular iron pool as a positive control, cells were treated with 100 μM ferrous sulfate (FeSO4; Sigma). As a negative control, cells were incubated with a 100 μM concentration of iron chelator DFO (Sigma, USA). The cells were washed and incubated with PG-SK in PBS at 37 °C for 10 min. Then, the cells were washed, incubated with DAPI to stain the nuclei and fixed by 4% paraformaldehyde fixation. Fluorescence images were then recorded automatically by a confocal microplate imaging reader at excitation 488 nm and emission 505 nm. Image acquisition was examined by confocal microscopy (Olympus FV1000).

### Western blot

Cells were lysed and the protein concentration of collected lysates was measured by BCA Protein Assay Kit (Pierce. USA). Then 50 μg lysates were separated by 12% SDS-PAGE gel and transferred onto PVDF membranes (Millipore, USA). The PVDF membranes were blocked in TBST containing 2% BSA for 1 h, antibody specific for TfR, GAPDH, Ferritin, P53, E2F3 and E2F1 were used. The antibodies were all from Cell Signaling Technology (MA, USA). Protein bands were detected by the enhanced chemiluminescence (ECL) reaction (Kibbutz Beit Haemek, Israel) and subjected to Alpha Innotech Flour Chem-FC2 imaging system (Alpha Innotech).

### Immunohistochemistry

Immunohistochemistry (IHC) was performed as described previously^55^. IHC was carried out with antibodies primary antibodies included TfR, GAPDH, Ferritin, P53, E2F3 and E2F1. Negative control sections were incubated with PBS instead of primary antibodies.

### Immunofluorescence and confocal microscopy

Briefly, cells were fixed in 4% paraformaldehyde and permeabilized with 0.5% Triton X-100 for 15 min. The cells were washed with PBS, blocked with 5% BSA in PBS for 1 h and incubated with primary antibody overnight at 4 °C. Ferritin was detected using a polyclonal antibody against FTL (CST, USA), then incubated for 1 h with the secondary antibody conju-gated with Alexa Fluorescence 568 (1:1000, Invitrogen) in 37 °C. DAPI was used to stain Nuclei (1:5000, Sigma). Cells were visualized by confocal fluorescence microscopy Transient transfection and small interfering RNA (siRNA).

P53 specific siRNAs were purchased from RiboBio (Guangzhou, China). The siRNA duplexes are 5′-CTACATGTGTAACAGTTCCUU-3′and p53 5′-GGAACTGTTACACATGTA GUU-3′. RNAi negative control was used as a Negative Control (NC). The miRNA mimics were synthesized by RiboBio (Guangzhou, China). Transfection were performed using Lipofectamine 2000 (Invitrogen, Carlsbad, CA, USA) recommended by the manufacturer.

### Statistical Analysis

All results are presented as means ± S.E.M of at least three independent experiments. Student’s t test, Mann-Whitney U test and Log-rank test were used to assess differences between two groups. A value of P < 0.05 was considered to be statistically significant.

## Electronic supplementary material

Magnetic field exposure system. Fig. S1
