# Supplementary material for: LF-MF inhibits iron metabolism and suppresses lung cancer through activation of P53-miR-34a-E2F1/E2F3 pathway
Source: Sci Rep. 2017 Apr 7;7:749. doi: 10.1038/s41598-017-00913-2 (PMC5429732; doi:10.1038/s41598-017-00913-2)
Supplement: Supplementary file 1 — Magnetic field exposure system. Fig. S1 [file 41598_2017_913_MOESM1_ESM.pdf]

LF-MF inhibits iron metabolism and suppresses lung cancer through activation of P53-miR-34a-E2F1/E2F3 pathway

Jing Ren, Liang Ding, Qianyun Xu, Guoping Shi, Xiaojing Li, Xiujun Li, Jianjian Ji, Dongya Zhang, Yaping Wang , Tingting Wang, Yayi Hou

Magnetic field exposure system. *Fig.S1*

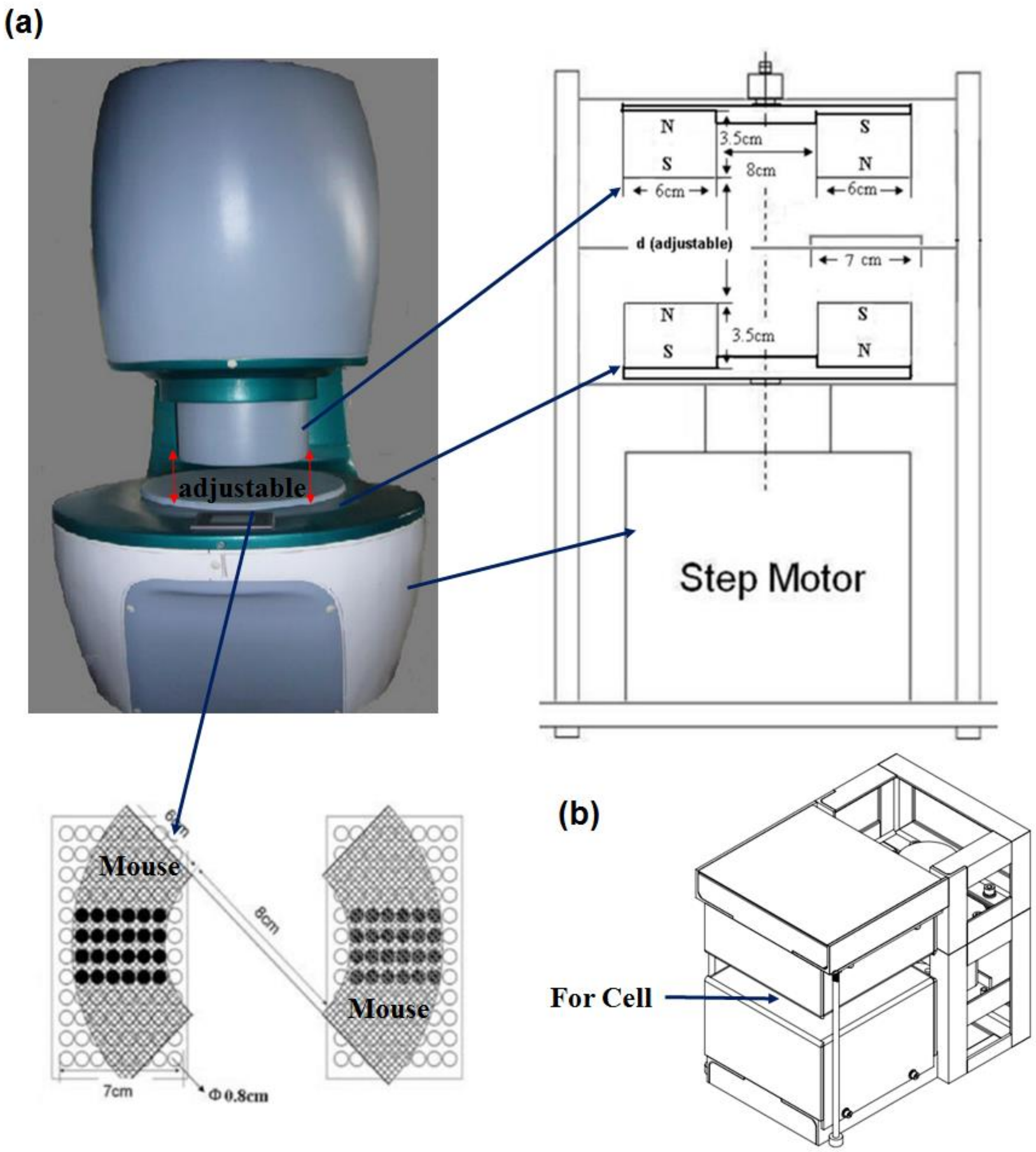

Fig.S1. Magnetic field exposure system.

(a) Instrument of magnetic field exposure system. Two pairs of fan-shaped NdFeB permanent magnets were arranged to establish magnetic fields. MF at the target site is alternative pulses with a maximum flux density of about 0.4 T. (**b)** The smaller instrument with similar structure to generate 0.4 T and 7.5 Hz LF-MF installed in Thermo Scientific Forma Series II 3120 Water-Jacketed CO_2 Incubators.
